# Social Networks and Mobile Applications Use in Young Patients With Kidney Disease

**DOI:** 10.3389/fped.2019.00045

**Published:** 2019-04-03

**Authors:** Raymond N. Haddad, Chebl C. Mourani

**Affiliations:** ^1^Department of Pediatrics, Hotel-Dieu de France University Medical Center, Saint Joseph University, Beirut, Lebanon; ^2^Division of Pediatric Nephrology, Department of Pediatrics, Hotel-Dieu de France University Medical Center, Saint Joseph University, Beirut, Lebanon

**Keywords:** social networking sites, mobiles applications, social media, kidney disease, pediatrics

## Abstract

**Objective:** To evaluate the use and benefits of social networking sites (SNS) and mobile applications (MA) in young patients with kidney disease (KD).

**Background:** Pediatric KD is prevalent. The Internet is increasingly being used to communicate rapid healthcare information to children about acute and chronic diseases with greater medical care satisfaction. There is a lack of data on social media (SM) utility in pediatric KD.

**Materials and Methods:** A descriptive, observational, and cross-sectional study was conducted on a national level. Data were collected from 4 different centers through a reviewed questionnaire.

**Results:** 83.9% of the 428 participants were Lebanese. The average age was 11.4 years (±7.1). 69.9% had chronic KD out of which 17.4% had undergone a kidney transplant while 9% were on dialysis. 69.6% of the participants affirmed the need of SM for the health of the sick child while only 9.8% are participating in a scientific forum and 4.7% used SM to find a potential organ donor. Some study variables were statistically associated with the participants' age, nationality, and stage of KD.

**Conclusions:** SM is important for the support and management of pediatric KD. We believe that SNS and MA will play a leading role in the lives of our patients in the upcoming future and will push the physician to be an active participant in the evolution of communication networks. To identify the efficacy of SM in enhancing communication between patients and health professionals, further stratified studies are needed.

## Introduction

Nowadays, social networking sites (SNS) and mobile applications (MA) are an integral part of everyday life for millions of users around the world (1, 2). More specifically, multiple online platforms and virtual communities are increasingly being used to communicate to young patients rapid healthcare information on several chronic diseases (3–5). Furthermore, a recent meta-analysis showed the major positive effect of SNS interventions on health behavior-related outcomes (6).

On the other hand, chronic kidney disease (CKD) is a major health problem worldwide. Although relatively uncommon in children, it may be an evolutionary and devastating disease with long-term consequences (7). The type of information searched by this specific population depends on their age and their ability to find it (8). However, only few SNS with sufficient reliability and quality of provided information are concerned in pediatric kidney disease (KD) (9, 10). For that, medical MA must be developed rigorously to ensure a reliable therapeutic complement for children with chronic diseases while maintaining a certain level of respect for confidentiality (11–13). With the lack of research on the use and benefits of SNS and MA for young patients with KD, we decided to conduct this study on a national level.

## Materials and Methods

### Patient Selection

**Figure 1:**
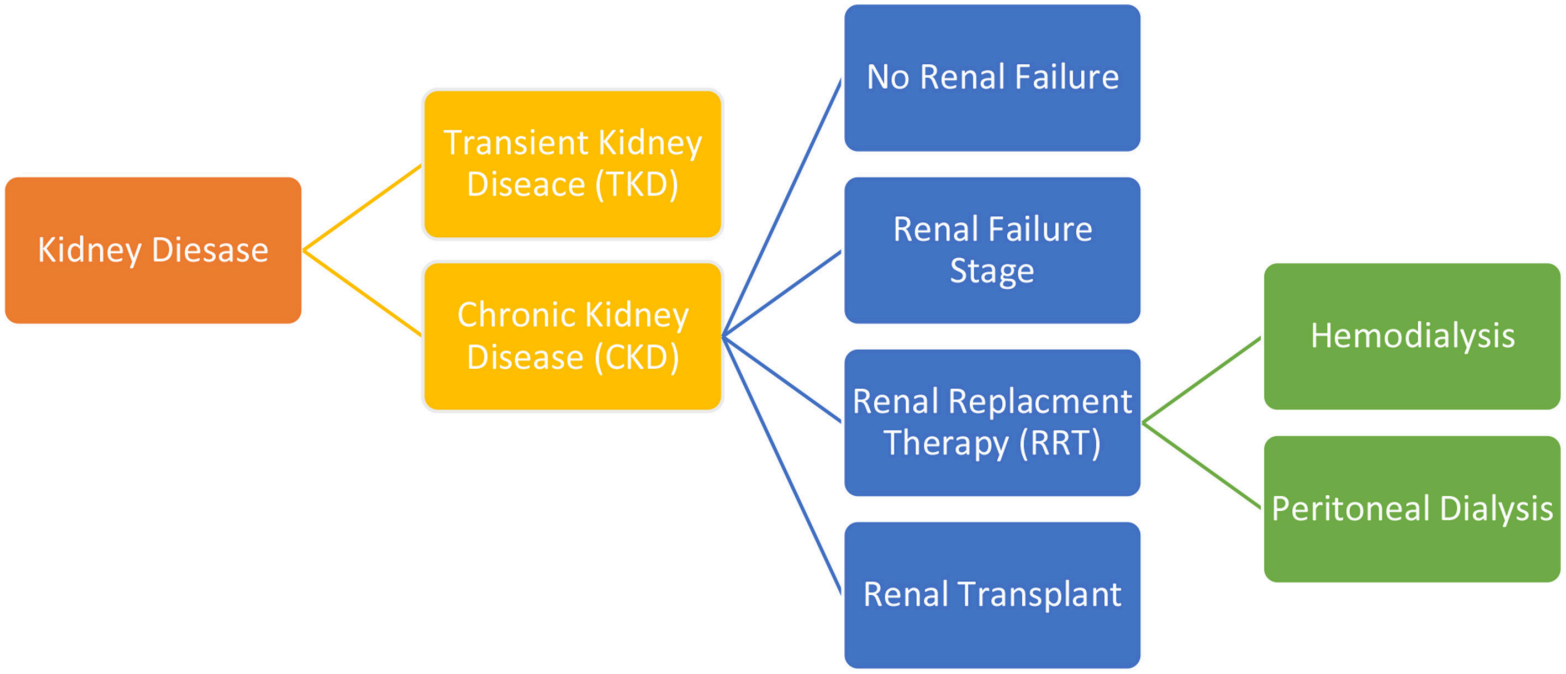
Patients study groups.

This is a descriptive, observational and cross-sectional study that was conducted over a period of three months (between July 2018 and September 2018), and received the approval of the ethical committee. During this period, data was collected from four different centers through a questionnaire distributed to young patients with KD, attending the clinics of seven Lebanese pediatric nephrologists. A written informed consent was obtained prior to participants' enrollment. Depending on the age of the child, our assistants were distributing version 1.0 of the questionnaire (QV1) to the parents of children aged <15 years old. If the patient was older than 15 years old, version 2.0 of the questionnaire (QV2) was distributed and could be completed by the patient himself, with or without the help of his parents. The two versions of the questionnaire included the same 23 questions (but addressed differently according to participant's age) and evaluated patients' socioeconomic status, demographic characteristics, major information about his KD, and about the use of the SNS and MA. Social status was evaluated according to the family highest educational level and parents' current occupation while economic status was evaluated according to family's monthly income. The two versions of the questionnaires were written in Arabic language to be understood by the majority of the participants. In the case of an illiterate parent, the doctor or the assistant completed the questionnaire after reading the questions to the participants. The anonymous questionnaires were then collected by authors who checked the accuracy of the answers and analyzed data collectively. Patients were then divided into 6 groups according to the stage and the type of the KD (Figure 1). At first, patients were divided according to the stage of the KD. The first group consisted of participants with a transient kidney disease (TKD) (treatable within a few weeks to a few months) and the other group included participants with CKD (who have been treated for a minimum of 12 months). The CKD group was then divided into 4 subgroups. The second group consisted of stage 1 CKD patients. The third group consisted of patients with renal failure (RF). The fourth group consisted of patients under renal replacement therapy (RRT) and was subdivided into 2 groups: hemodialysis or peritoneal dialysis. The final group consisted of patients who underwent renal transplantation.

### Statistics

Discrete variables were summarized as percentages and continuous variables as mean with standard deviation. Statistical analysis of the categorical variables was conducted using Fisher's exact test and by *t*-test for continuous variables. Statistical analyses were computed using SPSS Statistics, version 21 for Mac (IBM, Armonk, NY), with a *P*-value < 0.05 considered statistically significant. All reported *P*-values are two-sided.

## Results

Rate of participation was 99.8%. One patient did not participate in the study because he was not interested in the subject. A total of 428 participants were included in the study. The average age was 11.4 years (± 7.1). The majority of the participants (54.4%) were male and Lebanese (83.9%). Only 10 participants (2.3%) were illiterate and needed assistant's help. Out of the 69 foreign participants, 51% were Syrians, 27% were Palestinians, 19% were Iraqi, and 3% were Egyptians. Almost three-quarters of the participants had a middle social status (73.1%) and 63.1% had a middle economic status. Concerning the place of residence, participants were equally distributed among Lebanon's five governorates. Socio-demographic characteristics of the participants are summarized in Table 1. Regarding the stage and the type of the renal disease, only a third of our participants had a TKD (30.1%). Among those with CKD (*n* = 299, 69.9%), 48.2% of the participants had stage 1 CKD, 25.4% had RF, 17.4% had undergone a kidney transplant while 9% were on RRT.

**Table 1:** Demographic characteristics and study groups.

	***N* (%), *n* = 428**
Age (years), M ± SD (range)	11.4 ± 7.1 (0.1–37)
Lebanese nationality, *N* (%)	359 (83.9)
Male, N (%)	233 (54.4)
**SOCIAL STATUS**, ***N*** **(%)**	
Low	95 (22.2)
Middle	313 (73.1)
High	20 (4.7)
**ECONOMIC STATUS**, ***N*** **(%)**	
Low	148 (34.6)
Middle	270 (63.1)
High	10 (2.3)
**PLACE OF RESIDENCE**, ***N*** **(%)**	
Beirut	79 (18.5)
Mount-Lebanon	106 (24.8)
South of lebanon and nabatiyeh	105 (24.5)
North of lebanon and akkar	64 (15)
Bekaa, Baalbek, and Hermel	74 (17.3)
**RENAL DISEASE GROUPS**, ***N*** **(%)**	
Transient renal disease	129 (30.1)
Chronic renal disease	299 (69.9)
Stage 1 chronic kidney disease	144 (33.7)
Renal failure	76 (17.8)
Hemodialysis	15 (3.5)
Peritoneal dialysis	12 (2.8)
Renal transplant	52 (12.1)

*M ± SD, Mean ± Standard deviation*.

**Figure 2:**
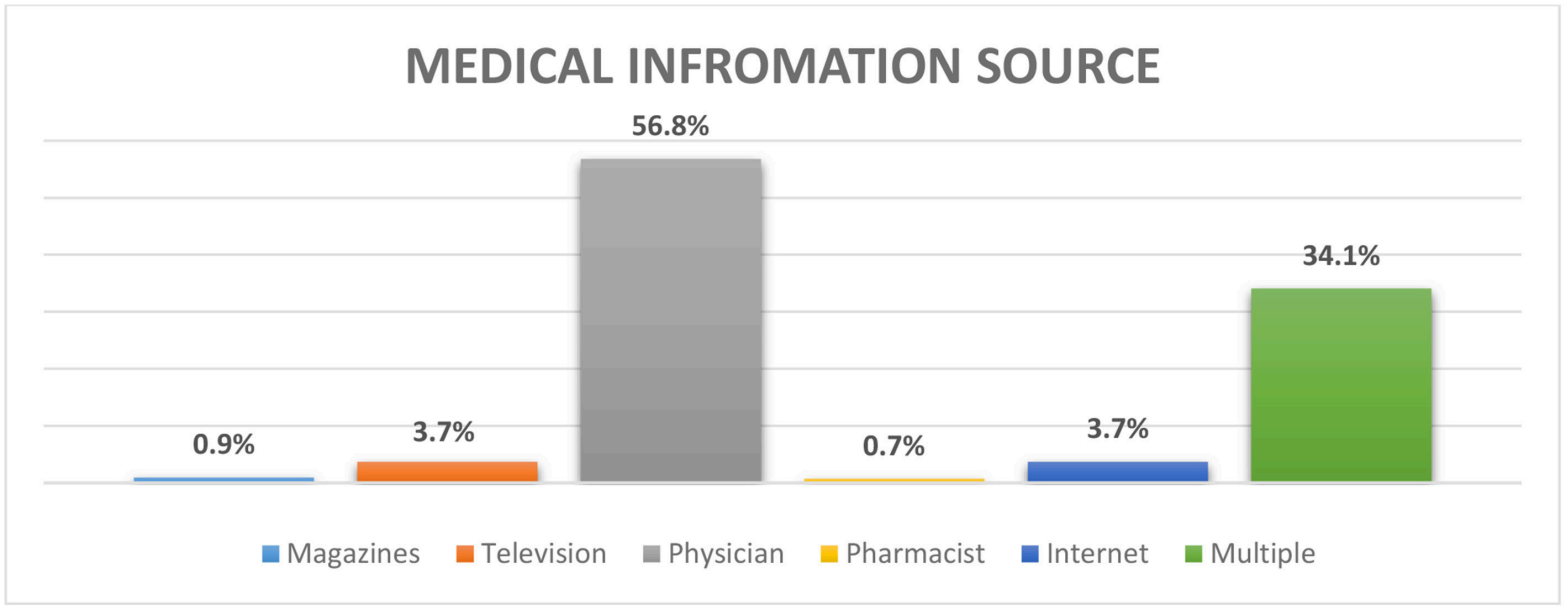
Distribution of different sources of medical information about the disease and its treatment among our participants.

Different sources of medical information used among our participants are summarized in Figure 2. Results show that 56.8% of the participants collect medical information related to their disease and its treatment from their physician while 34.1% of the participants rely on multiple sources of information.

Distribution of positive responses to the 9 different questions evaluating the use of social media (SM) is summarized in Table 2. Results showed that 69.6% of our participants confirmed the need of SNS and MA for the health of the sick child, 37.1% used short message service (SMS) to communicate with their physician. On the other hand, only 9.8% of patients were participating in a scientific forum and 4.7% used SM to find a potential organ donor.

**Table 2:** Positive responses to the different items of the survey questionnaire.

	***N* (%)**
Necessity of SNS and MA for the health of the sick child, *n* = 411	286 (69.6)
SNS and MA use to recognize the disease and its treatment, *n* = 428	82 (19.2)
SNS and MA use to communicate with specialists, *n* = 423	115 (27.2)
Participation in a scientific forum, *n* = 419	41 (9.8)
E-mail box use to communicate with the doctor, *n* = 424	67 (15.8)
SMS use to communicate with the doctor, *n* = 423	157 (37.1)
SMS use to make an appointment, *n* = 423	160 (37.8)
Communicate with kidney disease patients through FB, *n* = 426	32 (7.5)
SNS and MA use to find a possible organ donor, *n* = 424	20 (4.7)

*SNS, Social Networking Sites; MA, Mobile Applications; SMS, Short Message Service; FB, Facebook*.

We then decided to determine whether there are any significant associations between the 9 questions evaluating the SM usage and the following variables (age, nationality, stage of KD, and KD group) in our study.

We found a statistically significant association between the age of participants and some variables of the study. In fact, it seems that older patients use more SM to recognize their disease and its treatment (14.8 ± 7.3 > 10.6 ± 6.8, *p* < 0.001) and use more Facebook application to communicate with other KD patients (15.9 ± 7.3 > 11 ± 7.0, *p* < 0.001), but they use less SMS to communicate with their physician (10.4 ± 6.6 < 12.1 ± 7.3, *p* = 0.027). It seems that the Lebanese participants use more Facebook application to communicate with other KD patients (96.9 > 82.7%, *p* = 0.043) whereas foreign participants use more the e-mail service (25.4 > 14.3%; *p* = 0.029) and the SMS (23.6 > 12%; *p* = 0.003) to communicate with their physician. There is a statistically significant association between the stage of the KD and some variables in the study. As a result, it appears that participants with CKD, when compared to those with TKD, use SM more frequently to recognize their disease, and its treatment (79.3 > 67.6%, *p* = 0.044), use more Facebook application to communicate with other patients with renal disease (90.6 > 68.5%, *p* = 0.008) and communicate through SM with a potential organ donor (95 > 69.1%, *p* = 0.011).

Finally, Table 3 above shows that there is a statistically significant association between the different groups of CKD and certain variables of the study. Indeed, it appears that participants undergoing dialysis use SM at the lowest rate to recognize their disease and its treatment (*p* = 0.006). In addition, participants with non-dialysis CKD use Facebook application at the highest rate to communicate with other KD patients (*p* < 0.001). Finally, patients undergoing dialysis or who underwent renal transplantation used SM to find a possible organ donor (*p* < 0.001).

**Table 3:** Distribution of participants' positive answers to the different study variables according to the study groups.

						**F^**a**^**	**p-value**
	**Gr1**	**Gr2**	**Gr3**	**Gr4**	**Gr5**		
Necessity of SNS and MA for the health of the sick child, *n* = 286	83 (29.0)	88 (30.8)	60 (21.0)	19 (6.6)	36 (12.6)	8.17	0.084
SNS and MA use to recognize the disease and its treatment, *n* = 82	17 (20.7)	21 (25.6)	19 (23.2)	8 (9.8)	17 (20.7)	14.34	0.006
SNS and MA use to communication with specialists, *n* = 115	42 (36.5)	29 (25.2)	18 (15.7)	9 (7.8)	17 (14.8)	7.29	0.119
Participation in a scientific forum, *n* = 41	16 (39)	8 (19.5)	8 (19.5)	3 (7.3)	6 (14.6)	4.88	0.287
E-mail box use to communicate with the doctor, *n* = 67	18 (26.9)	22 (32.8)	11 (16.4)	4 (6.0)	12 (17.9)	2.64	0.621
SMS use to communicate with the doctor, *n* = 157	51 (32.5)	53 (33.8)	25 (15.9)	10 (6.4)	18 (11.5)	0.94	0.924
Communication with kidney disease patients through FB *n* = 32	3 (9.4)	5 (15.6)	10 (31.3)	5 (15.6)	9 (28.1)	22.72	<0.001
SNS and MA use to find a possible organ donor, *n* = 20	1 (5.0)	2 (10.0)	3 (15.0)	7 (35.0)	7 (35.0)	31.48	<0.001

a *Fisher test*.

*SNS, Social Networking Sites; MA, Mobile Applications; SMS, Short Message Service; FB, Facebook; Gr1, Transient Renal Disease; Gr2, Stage 1 CKD; Gr3, Renal Failure; Gr4, Dialysis; Gr5, Renal Transplant*.

## Discussion

On the first hand, CKD is a major example of chronic illness in childhood (7). It causes psychological tensions and a low health-related quality of life for both parents and sick children (14). Pediatric CKD is usually diagnosed early in life. The most common complications encountered in this population are infections, bone diseases, poor growth, and kidney function impairment. Despite the possible interventions such as RRT or kidney transplantation, mortality rate remains 30 times higher than the one of healthy children (7). Parents of CKD children have to deal with complex treatment schedules including nutritional restrictions, and invasive procedures such as hemodialysis or peritoneal dialysis. They also put on a lot of efforts to manage this complicated situation (14). They have to act like nurses, pharmacists, and doctors in addition to the burden of their usual parenting responsibilities. In order to help the family cope with the difficulties encountered in all stages of this devastating disease, ongoing communication and support, personalized care, and continuous information are needed.

On the other hand, the current range of SM platforms facilitates exchange of information and makes professional networking possible. Multidisciplinary virtual communities have great potential to facilitate the transfer of experimental and research knowledge by breaking down professional and organizational boundaries (15). The use of SNS dedicated to acute and chronic health problems are gaining ground with the families of a chronically ill children, with a positive impact on the received medical information about the disease and its treatment (6, 16). Young patients must be involved in the development of all resources related to the management of their long-term conditions to ensure that all their needs and expectations are met (17).

Our study was conducted to evaluate the use of SNS and MA among young patients with KD and it is the first of its kind in Lebanon. To our knowledge, no similar studies have been conducted in the Arab world and North Africa up to this date. The questionnaires were distributed to the seven most well-known pediatric nephrologists practicing in Lebanon and representing the four out of five most recognized Lebanese university hospital centers. This gives the study an important national value. Although the seven physicians participating in the study practice in the capital Beirut and its suburbs, our study participants come equally from the major five governorates of the country. The 69 foreign participants constitute 17% of our participants and surprisingly represent the equivalent proportions to the figures given by the latest statistics of the United Nations Refugee Agency (UNHCR), which estimates the number of registered Syrians refugees at 976,002, Iraqi at around 100,000 and Egyptian at 83,312 for a total Lebanese population of around 5,850,000. Around 450,000 Palestinian refugees were registered in Lebanon with the United Nations Relief and Works Agency for Palestine Refugees in the Near East (UNRWA) in July 2014.

It appears in our study that the physician is the main provider of medical information for patients and their families. In fact, young patients always refer to the pediatric nephrologist for any medical, surgical, pharmacological, or even psychological intervention. He must help the family by providing specialized counseling services and must supervise a continuous multidisciplinary education while giving complete explanations about the child's illness. Unfortunately, there is a lack of research evaluating how health professionals individually and collectively provide support to the family of a chronically ill child. In this spectrum, a multicenter study conducted in Great Britain highlights the educational role of parents in a national nephrology network through testimonials from multidisciplinary learning members. It described how 112 professionals recognized each other's roles, shared their expertise and worked in permanent collaboration while offering education to parents (18).

In our study, 69% of participants and their parents recognized the importance of SNS and MA for the health of the sick child. In fact, thousands of internet platform promote interactions between health professionals during their clinical practice, broaden their professional links and enhance medical education and training (15). However, these benefits come with some limitations, as the knowledge of the technical requirements for the use of digital technology as well as the maintenance of professionalism and data protection. Further research to evaluate the potential and the efficacy of SNS in enhancing communication between medical professionals are needed (15). In our study, 19.2% of participants use SNS to recognize the disease and its treatment. They clearly understand digital security and search for information. The ease of access to online medical sites is possible for parents as well as for young patients who are digital natives and can understand the advantages and disadvantages of using web resources. In fact, many MA are currently offering a digital support and useful information to accomplish better symptoms control for patients at the different stages of their chronic disease (13). Another review of the literature identified 19 publications on MA used for chronic illness in adolescents (12). Rigorous research into the potential of MA will optimize health care delivery and its outcomes (19). For that, the premise is to develop a MA based on scientific evidence that increases children ability to manage their disease with confidence while meeting his assistance needs and optimizing the expected results. Laranjo and al conducted one of the first meta-analysis concerning this subject, and affirmed that interventions on SNS appear to be effective in promoting changes in health behaviors. Further research studies on the application of these promising tools are needed (6).

We note that 37% of participants in our study use SMS to make an appointment and the same percentage of participants uses the SMS also to communicate medical data and receive medical information. In fact, SMS reminders on mobile phones increase attendance rates at healthcare appointments in comparison to non-reminders and postal reminders and are more cost effective than phone call reminders (20). They may also have some potential to reduce serious adverse events leading to hospitalization. In some other cases, SMS may provide benefit in supporting the self-management of long-term diseases. However, no user safety perception, health outcomes, or adverse effects of the intervention were reported and there are remarkable information gaps regarding the acceptability, long-term effects, risks, and costs of such interventions (21). On the other hand, little evidence on the acceptability of the SMS was found to support the self-management of long-term conditions. In one study, the majority of participants expressed their willingness to continue using this intervention (22). Nevertheless, another paper suggested that, over time, interest for this type of support gradually decreases (23). For that, the short- and long-term acceptability of the SMS for disease self-management need to be well studied.

In our study, only 15.8% of the participants communicated with doctors by email. We found a significant difference in favor of the foreign population using the email in a higher rate when compared to Lebanese participants. A Lebanese study noted that email communication between doctors and patients has promising advantages in terms of reducing health care costs as well as improving health promotion and disease management yet, its use among physicians is still insufficient (24). There is also a gap between the will of physicians and the actual practice of e-mail communication. These differences can be explained by several factors related to the physicians: some doctors are not convinced that patients appreciate, need and are able communicate by email; others are still waiting for strong evidence of this service performance and effectiveness. We believe that governmental agencies should guide and promote e-mail communication to progress as a means of widespread communication in the near future.

Finally, Facebook is the most popular global platform for internet users. Only 75% of our patients and parents use it to communicate with other KD patients, particularly the chronically ill ones in a statically significant way. The majority of adolescent patients do not use SM to connect with other people with similar conditions and they do not use the internet to find information about their diagnosis (25). In fact, most teens do not disclose their personal health information on SNS, although Van der Velden et al. study found widespread use of Facebook. In fact, according to kids and adolescents, Facebook is seen as a place where you have to be “normal” and cool rather than “a sick teenager”. It is a place where teenagers stay in touch with each others and it is not seen as a place to discuss their diagnosis and treatment. Our participants also affirm that they get involved in scientific forums related to their disease. While medical forums are currently widely used by medical staff, patients find many interesting forums and even online medical support programs offered by specialized teams in pediatric nephrology. Current data on the use of SM by health professionals suggest that virtual communities are considered as knowledge portals where craft knowledge is exchanged. Rolls and al point out in their study that only a limited number of publications regarding new SM platforms are available (3).

## Conclusion

Through this multicenter study in which 71% of the participants were recruited from our center, we believe that SM will play a leading role in the lives of our patients in the upcoming future. Nowadays patients are becoming more demanding and seek fast medical answers and explanations through SM. We encourage them to share their SM use experiences with each other in the clinic waiting room. The role of the treating physician is to be at the center of the action by guiding their patient web surfing and correcting misinformation. It seems essential to us that the physician, and in particular, the pediatric nephrologist realizes the impact of the current means of internet communication in his current practice. He could also participate if possible, in the making of a medical website in order to deliver the most accurate basic medical information. Furthermore, it is becoming a common practice to use text messaging and Whatsapp application in our daily practice to receive laboratory or radiological exams. One of the new tasks of our assistants is to respond in the shortest time possible to parents' and patients' questions to deliver through these applications the therapeutic modifications and medical advice especially to the patients living abroad. Finally, this study showed that, although the pediatric nephrologist is currently the center of the medical information, SNS and MA are starting to take a large place in the communication of medical information and force physicians to play an active role in the evolution of the different communication platforms.

## Ethics Statement

This study was reviewed and approved by Saint Joseph University Research Ethics Board.

## Author Contributions

RH analyzed the data, interpreted the results, and took the lead in writing the manuscript. CM conceived of the presented idea and supervised the project. All authors discussed the results.

### Conflict of Interest Statement

The authors declare that the research was conducted in the absence of any commercial or financial relationships that could be construed as a potential conflict of interest.
